# Decoding Accuracy in Supplementary Motor Cortex Correlates with Perceptual Sensitivity to Tactile Roughness

**DOI:** 10.1371/journal.pone.0129777

**Published:** 2015-06-11

**Authors:** Junsuk Kim, Yoon Gi Chung, Jang-Yeon Park, Soon-Cheol Chung, Christian Wallraven, Heinrich H. Bülthoff, Sung-Phil Kim

**Affiliations:** 1 Department of Brain and Cognitive Engineering, Korea University, Seoul, Republic of Korea; 2 IBS Center for Neuroscience Imaging Research, Sungkyunkwan University, Suwon, Republic of Korea; 3 Department of Global Biomedical Engineering, Sungkyunkwan University, Suwon, Republic of Korea; 4 School of Biomedical Engineering, Konkuk University, Chungju, Republic of Korea; 5 Department of Human Perception, Cognition and Action, Max Planck Institute for Biological Cybernetics, Tübingen, Germany; 6 Department of Human and Systems Engineering, Ulsan National Institute of Science and Technology, Ulsan, Republic of Korea; Birkbeck, University of London, UNITED KINGDOM

## Abstract

Perceptual sensitivity to tactile roughness varies across individuals for the same degree of roughness. A number of neurophysiological studies have investigated the neural substrates of tactile roughness perception, but the neural processing underlying the strong individual differences in perceptual roughness sensitivity remains unknown. In this study, we explored the human brain activation patterns associated with the behavioral discriminability of surface texture roughness using functional magnetic resonance imaging (fMRI). First, a whole-brain searchlight multi-voxel pattern analysis (MVPA) was used to find brain regions from which we could decode roughness information. The searchlight MVPA revealed four brain regions showing significant decoding results: the supplementary motor area (SMA), contralateral postcentral gyrus (S1), and superior portion of the bilateral temporal pole (STP). Next, we evaluated the behavioral roughness discrimination sensitivity of each individual using the just-noticeable difference (JND) and correlated this with the decoding accuracy in each of the four regions. We found that only the SMA showed a significant correlation between neuronal decoding accuracy and JND across individuals; Participants with a smaller JND (i.e., better discrimination ability) exhibited higher decoding accuracy from their voxel response patterns in the SMA. Our findings suggest that multivariate voxel response patterns presented in the SMA represent individual perceptual sensitivity to tactile roughness and people with greater perceptual sensitivity to tactile roughness are likely to have more distinct neural representations of different roughness levels in their SMA.

## Introduction

Humans perceive various kinds of mechanical stimuli when interacting with the external environment. In particular, discrimination of tactile roughness is essential in perceiving the material characteristics of a physical surface. Perceptual sensitivity to tactile roughness has been investigated by probing how physical properties of a surface are perceived by an individual’s somatosensory system [1]. Many psychophysical studies have revealed that perceptual roughness sensitivity to identical tactile stimuli varies across individuals, leading to large differences in roughness discrimination capability [2–6]. For example, Libouton and colleagues calculated individual discrimination thresholds to quantify perceptual sensitivity, and then examined inter-subject variability from a human population [2]. They reported that the perceptual differences between individuals were as high as 32.5 μm (particle size of sandpaper) while the average discrimination threshold was 43.5 μm. Moreover, multidimensional scaling (MDS) methods used to describe individual perceptual maps of tactile roughness revealed significant individual differences in roughness perception [6]. Despite a significant amount of psychophysical research, however, little attention has been devoted to investigate the neural substrates underlying such individual differences in perceptual sensitivity to roughness. In the present study, we set out to find which parts of the brain were implicated in the large individual differences in tactile roughness sensitivity.

To date, numerous neurophysiological studies have addressed the neural representations of tactile roughness information in the human brain by examining neuronal responses to stimuli with various degrees of roughness (for a review, see [7]). Specifically, posterior portions of the postcentral gyrus have been identified to be critical for tactile roughness discrimination as revealed by a functional magnetic resonance imaging (fMRI) study [8] and a positron emission tomography (PET) study [9]. Additionally, several studies have sought to demonstrate the relation between neural activity and behavioral responses in tactile discrimination tasks. Studies with single cell recordings in non-human primates have shown that neuronal firing activity in the somatosensory cortex is implicated in behavioral decisions [10, 11]. A human fMRI study found a relationship between prefrontal cortical activity and behavioral decisions during a tactile discrimination task [12]. Yet, none of these studies has explicitly examined the source of individual differences in tactile perception.

Here, we aim at finding a neural correlate of individual perceptual sensitivity to tactile roughness via a combination of psychophysical and fMRI-based neurophysiological approaches. In our study design, individual differences in behavioral performance during a tactile roughness discrimination task were compared with the corresponding neuronal activity of the same participants in follow-up fMRI experiments. In particular, we employed the multi-voxel pattern analysis (MVPA) method to investigate neural information of tactile roughness in the fMRI data. Because our data analysis focused on identifying brain regions exhibiting distinct neural activity patterns across roughness levels, a contrasting analysis using the classical general linear model (GLM) could not fully satisfy our purpose. We therefore considered the MVPA more appropriate to our study than the GLM analysis. Moreover, previous studies have revealed that the MVPA could relate distinct activity patterns within a certain brain region to stimulus parameters, and accumulate the weak information from each brain region in an efficient way [13, 14]. Several fMRI studies have used the MVPA to investigate neural tactile information processing [15, 16], mostly focusing on delineating the neuronal activation patterns in response to tactile stimulations. Liang and colleagues employed a multivariate pattern classifier to identify neural representations in the human brain elicited by vibrotactile stimuli on the fingers [15]. Along similar lines, a voxel-based principal component analysis (PCA) was used to investigate the functional neural networks related to tactile stimulus discrimination [16]. In the present study, we employed an information-based searchlight MVPA [17] to search for neural activity involved in perceptual sensitivity to tactile roughness.

We first searched for the brain regions exhibiting neuronal activity patterns associated with roughness discrimination using a whole brain searchlight MVPA. Once the brain regions producing significant decoding results were identified, we investigated whether the decoding accuracy within these brain regions correlated with individual behavioral performance of roughness discrimination. The behavioral performance was measured by the just noticeable difference (JND) of each participant [18]. The brain regions showing a correlation with the JND would most likely serve as a neural substrate underlying individual perceptual sensitivity to tactile roughness.

## Materials and Methods

### Participants and Ethics Approval

Sixteen healthy volunteers (six females, Koreans, 25.3 ± 3.8 years old, age range: 20–34 years) with no history of neurological disorders participated in the study after having given written informed consent. All were right-handed, with normal or corrected-to-normal vision. No participant reported having deficits in tactile processing. Experimental procedures were approved by the Korea University Institutional Review Board (KU-IRB-11-46-A-0), and the study was conducted in accordance with the Declaration of Helsinki.

### Tactile Stimuli

A previous study of fine-surface texture perception reported the absolute detection threshold of humans to be between 1 and 3 μm in particle sizes [19]. Moreover, Fechner’s law describes that the magnitude of a subjective sensation increases in proportion with the logarithm of the tactile stimulus intensity [20]. Based on these findings, we determined the grit values of abrasive papers for our experiments: the minimum particle size was set to 3 μm (corresponding to a grit value of 4000) and linearly increased on a log-scale. Five different roughness levels of aluminum-oxide abrasive papers (Sumitomo 3M Limited, Tokyo, Japan), which were validated in the aforementioned study [19], were used in our experiments. The grit values assigned by the manufacturer were 400, 1200, 2000, 3000, and 4000, corresponding to average particle sizes of 40, 12, 9, 5, and 3 μm, respectively. A smaller grit value or a larger particle size indicates a rougher surface.

In the behavioral tactile discrimination task, four out of the five abrasive papers were used (based on a preliminary study, the particle size of 40 μm was too easy to discriminate). Two abrasive papers sized 3 × 3 cm^2^ were attached 1 cm apart from each other on a plastic plate sized 5 × 9 cm^2^. Two abrasive papers with the same roughness could be positioned in a single plate. In total, 16 paired-stimulus plates were used in the behavioral experiments: counterbalanced pairwise combinations of four distinct stimuli, plus four stimulus plates having the abrasive papers with the same roughness (_4_P_2_ + 4 = 16). In the fMRI experiments, all five abrasive papers were used, each being attached on five stimulus plates. A single abrasive paper sized 3 × 3 cm^2^ was attached at the center of a plastic plate sized 5 × 5 cm^2^.

### Experimental Design

**Fig 1:**
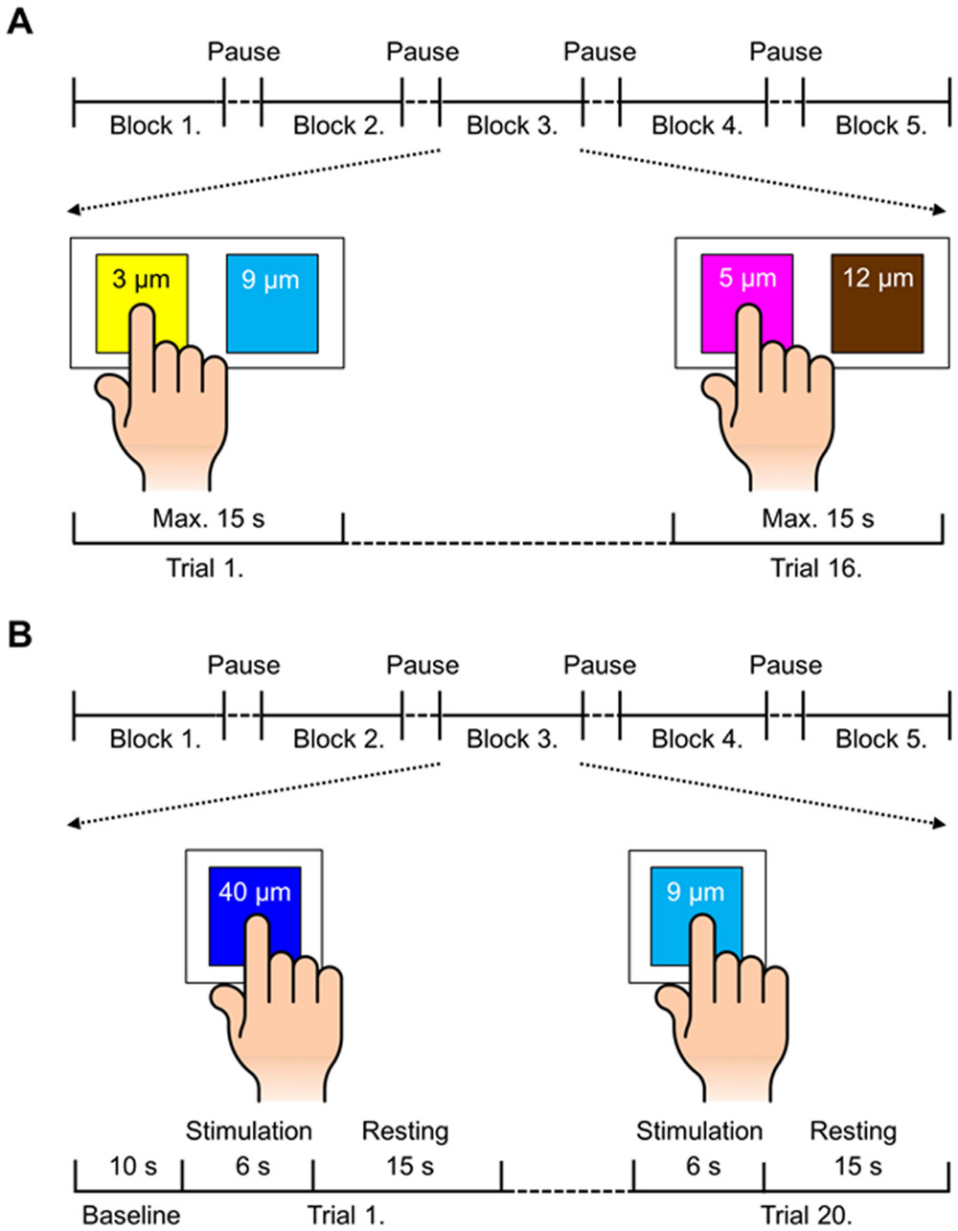
Structure and time course of the experimental design. (A) In the behavioral experiments, participants were asked to respond on which of the two stimuli felt rougher after active exploration with the tip of the right index finger. Each participant performed a total of 80 trials (16 trials × 5 blocks) and the maximum duration allowed per trial was 15 s. (B) In the fMRI experiments, the participants performed a total of 100 trials (20 trials × 5 blocks) and each trial included two periods: exploration for 6 s followed by rest for 15 s.

Prior to the fMRI scanning, participants performed the behavioral tactile discrimination task outside the MR room, and then moved into the MR room and participated in the fMRI experiment (Fig 1). Participants completed five blocks, each consisting of 16 trials (Fig 1A). The procedure of every block was identical except for the sequence of stimulus presentation. In each trial, participants explored two abrasive papers with the right index fingertip and reported verbally, which of them felt rougher. Participants were instructed to close their eyes during this discrimination task to block visual information of the stimuli. The duration of a single trial was 15 s and the inter-trial interval was set at 5 s. Participants were given 16 paired-stimulus plates in a pre-defined random order to ensure that every pair was presented for the same number of times. We used a function that generated a sequence of numbers pseudo-randomly to obtain random sequences. A 1-min break was provided between the blocks and the entire experiment took approximately 30 min.

During the functional image acquisition, participants lay in a supine position in a head-only MR scanner with their right arm comfortably placed on their upper abdomen and viewed the visual instruction screen on the MR-compatible head-mounted display goggle with a resolution of 800 × 600 pixels (Nordic ICE, NordicNeuroLab, Bergen, Norway). The stimulus plate with an abrasive paper was placed on the participants’ upper abdomen in a consistent manner by an experimenter. To replace the stimulus plates, an experimenter was positioned at the entrance of the magnet bore where he could easily reach out to the participants. The fMRI experiments consisted of five blocks, each with 20 trials; short breaks were provided for about 1 min between the blocks (Fig 1B). A single block started with a 10-s baseline period followed by a series of 20 trials. Each trial was composed of two consecutive periods: an exploration period of 6 s followed by a resting period of 15 s. A Korean word, ‘자극’ (‘Stimulation’ in English), and a fixation cross, ‘+’, were displayed at the center of the instruction screen to indicate the exploration and resting periods, respectively. The instruction to initiate the exploration was synchronized with the MR pulse sequences after baseline period in each block. During the exploration period, participants were instructed to explore the tactile surface using horizontal movements; specifically, participants moved their right index fingertip from side to side. After each exploration period, the participants slightly lifted their finger so as not to interfere with stimulus replacement; the experimenter replaced a set of the stimulus plates in a pre-defined order during each resting period. Next, during the resting period, the participants placed their finger on the upper abdomen. Visual instructions were preferred to auditory instructions because of the possibility that the loud sounds generated due to the rapid alterations of current in the MR scanner might hinder the clear perception of auditory cues. Since the image acquisition time was set at 3 s per volume (see Data Acquisition and Preprocessing), each trial resulted in 7 volumes for the 21 s duration. Over the entire experiment, 700 functional volumes (i.e., 5 blocks × 20 trials × 7 volumes per trial) were acquired in each participant.

### Data Acquisition and Preprocessing

Neuroimaging data were acquired using a 3T MRI system (Magnetom TrioTim, Siemens Medical Systems, Erlangen, Germany) equipped with a standard 16-channel head coil. Anatomical images were acquired using a T1-weighted 3D MPRAGE sequence with repetition time (TR) = 1,900 ms, echo time (TE) = 2.52 ms, flip angle = 9°, field of view (FOV) = 200 mm, and spatial resolution = 1 × 1 × 1 mm^3^. Functional images, covering the whole cerebrum, were acquired using a T_2_*-weighted gradient echo-planar imaging (EPI) sequence with 35 slices, TR = 3,000 ms, TE = 30 ms, flip angle = 90°, FOV = 240 mm, slice thickness = 3 mm, inter-slice gap = 0.75 mm, and in-plane resolution = 3 × 3 mm^2^. The preprocessing and statistical analysis of fMRI data were performed using SPM8 (Wellcome Department of Imaging Neuroscience, UCL, London, UK) and a high-pass filter of 128 s was used to eliminate low frequency noise. The EPI data were corrected for slice-timing differences, realigned for motion correction, co-registered to the individual T1-weighted images, normalized into the Montreal Neurological Institute (MNI) space, and spatially smoothed by a 4-mm full-width-half-maximum (FWHM) Gaussian kernel.

### Behavioral Data Analysis

To determine the relation between a stimulus and its perceptual variance, we designed the experiment to measure the relative perceptual sensitivities in individuals. We set the grit value of a left-side abrasive paper on a stimulus plate as a reference value (i.e., one of the four values: 1200, 2000, 3000, and 4000), and then defined relative difference in roughness compared to the reference paper. Instead of using a fixed standard stimulus surface as reference, different reference surfaces were presented to participants for each trial. This might lead to unexpected variance or bias to the data because participants did not perceive roughness in a linear scale. However, as a basic objective of this study was to examine individual differences in perceptual roughness sensitivity, we focused on individual abilities of detecting relative differences of roughness rather than the exact different thresholds of presented roughness. The difference between the grit value of a right-side abrasive paper and the reference value was calculated, resulting in one of 11 difference levels (±2800, ±2000, ±1800, ±1000, ±800 and 0). We defined the relative roughness of the right-side paper by setting no difference as 100%, and -2800 and 2800 as 50% and 150%, respectively. The intermediate degrees of relative roughness were determined according to the difference levels. For each degree of relative roughness, we obtained a ratio of the number of trials in which a participant reported that the right-side paper was rougher to the total number of trials with a given degree of relative roughness. A psychometric function was then fitted to these ratio data against their relative roughness to assess behavioral discrimination sensitivity using a bootstrap method (the psignifit toolbox for Matlab; [21]). A fitted psychometric function showed a cumulative probability that the right-side abrasive paper was perceived to be rougher than the reference paper, as a function of its relative roughness. Therefore, a steeper slope indicated more sensitive discriminability. Here, discrimination sensitivity of each participant was measured as a difference of roughness intensity values between the 25_th_ and 75_th_ percentiles. This difference is known as the just noticeable difference (JND).

For the reliability of the JND, we estimated each participant’s goodness of fit value, in which a bad fit indicated a low reliability of the JND estimate. We fitted the logistic function to the psychometric curve and the individual deviances were evaluated to measure goodness of fit [22]. A 95% confidence interval was calculated based on the simulations from the bootstrapping procedure (n = 999). If the observed deviance was outside the 95% confidence limit, we considered the participant’s data as an outlier.

### Functional Imaging Data Analysis

Functional image data was analyzed using the searchlight MVPA [17]. In particular, we utilized parameter estimates (i.e., model coefficients) extracted from a GLM for the searchlight MVPA. Parameter estimates explained how much the stimulus location variable contributed to the variation of neuronal signals. They have been employed as input features to the searchlight MVPA in previous studies [23, 24]. A standard predictor was built by the convolution of a box-car function of the stimulation ‘on’ periods with a standard model of the hemodynamic response function (HRF) of SPM8. We implemented a GLM independently for each stimulation condition without averaging across trials to increase the number of exemplars used for training the classifier. Therefore, a total of 100 regressors (5 roughness levels × 4 trials per block × 5 blocks) were acquired for each participant. Regressors were fitted to each voxel and the resulting parameter estimates were used as input features to the MVPA. A searchlight with a 7×7×7 voxel cube, which contained the activation patterns of a maximum of 343 voxels surrounding each voxel, scanned the whole brain volume. For each cube, a Gaussian Naïve Bayes (GNB) classifier was used to decode the five different levels of roughness from a multi-voxel activation pattern. The classification accuracy resulting from a 5-fold cross-validation method in a leave-one-block-out paradigm (i.e., each experimental block was considered as one fold) was stored along with the coordinate of the central voxel of the cube. The accuracy value stored for each voxel was corrected by subtracting chance-level accuracy (0.2 in this case, recall that the classifier predicted one out of five different roughness levels) to yield deviations from chance (S1 Dataset). Using these data, we generated each participant’s (spatially-normalized) brain mask of decoding accuracies. A random-effects group analysis was performed on the single-subject accuracy masks to establish commonalities among individual neural decoding results. A one-sample t-test against 0 was applied to verify above-chance decoding accuracy for every voxel.

To correct the searchlight cluster results for multiple comparisons, we employed the method described by Oosterhof and colleagues [25]. We compared the size of the clusters resulting from the group analysis to a reference distribution of clusters that one would obtain by chance. If there is no real effect, the sign of the searchlight accuracy values would be ‘+’ or ‘-’ with an equal probability of 50% (which is allowed under the null hypothesis of chance accuracy). To identify how large clusters would be determined when the null hypothesis is true, we sampled from the searchlight results maps and randomly flipped the sign of the maps of a random number of participants. These maps were then considered as one group sample from the null effect case, and a random-effect analysis on these maps calculated the size of the biggest cluster. This procedure was repeated 1000 times and the computed cluster sizes for each iteration were collected, yielding the distribution of cluster sizes under the null hypothesis. In this study, we reported the clusters in the 5% of the upper tail (i.e., p < 0.05 corrected for multiple comparisons via cluster size).

Additionally, a univariate GLM group analysis was performed to search for brain regions associated with blood-oxygen-level-dependent (BOLD) signal differences between the tactile roughness exploration and the resting conditions on each degree of roughness. The resulting contrast images for each participant were entered into a random-effects group analysis. This univariate analysis was conducted to confirm that the neuronal activation patterns in our data corresponded to established patterns in the previous literature and contained adequate information for the multivariate decoding analysis.

### Correlation Analysis

To determine a correlation between perceptual and neural discriminative patterns of tactile roughness, we correlated the JND values with the decoding accuracy values for each significant cluster. In particular, we estimated the robust regression coefficients using a modified least-squares linear method to reduce the effects of outliers. The significance of the correlation coefficient was evaluated with the F-test. Since a smaller JND value indicates a higher perceptual sensitivity, a negative correlation (higher decoding accuracy with smaller a JND) indicates that the multivariate neuronal activity patterns in the examined cluster capture behavioral performance in our roughness discrimination task. Moreover, to probe the influence of non-linear roughness perception (recall that we had used tactile surfaces with non-linearly scaled roughness values, thus percentage of correct answers can be a complementary behavioral measure), we performed an additional correlation analysis between decoding accuracy and percentage of correct answers.

## Results

### Behavioral Data Analysis

Table 1 summarizes the JND values, goodness of fit values (i.e., deviance), and percentage of correct answers for all 16 participants. Examples of the psychometric function are shown in S1 Fig. In 13 participants, the fitting procedure resulted in acceptable goodness of fit statistics. However, the behavioral data from three participants (# 6, 7, and 10) were not fitted well and we therefore excluded from the further analyses. The mean and the standard error values in the table were calculated excluding the data of participants 6, 7, and 10.

**Table 1:** A summary of the behavioral experiments.

Participant	JND	Correct answer (%)	Goodness of fit (deviance)
P1	37.59	81.67	12.83
P2	40.20	80.00	7.15
P3	35.27	78.33	14.74
P4	21.83	58.33	3.94
P5	87.52	80.00	6.33
P6*	690.49	45.76	22.52
P7*	-213.28	48.33	19.12
P8	39.99	75.00	10.33
P9	83.92	83.61	9.19
P10*	152.92	88.33	24.45
P11	86.52	80.00	4.62
P12	31.56	65.00	8.57
P13	26.02	58.33	8.13
P14	32.42	61.67	5.41
P15	30.11	65.00	6.92
P16	109.74	85.00	11.31
Mean	50.98	73.23	8.42
Std. Error	8.18	2.77	0.89

* Data from participants 6, 7, and 10 were considered as outliers and excluded from the analysis.

### Functional Imaging Data Analysis

**Fig 2:**
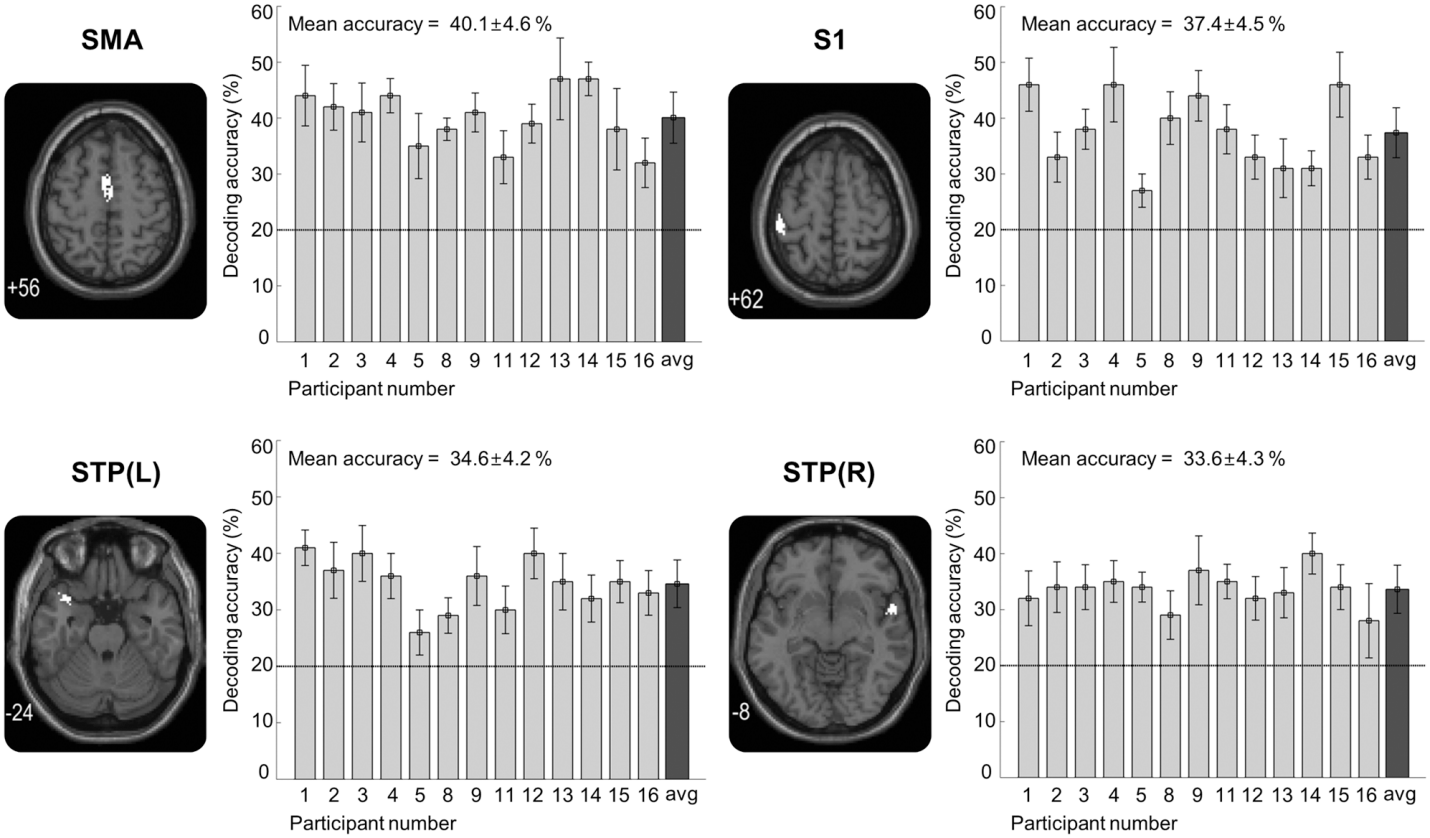
Results of the whole brain searchlight MVPA. Searchlight analysis identified four brain regions showing significant decoding performance in the prediction of five different levels of roughness. For each region, the left panel shows a sagittal slice of the brain (Z-coordinate of slice indicated in bottom left corner). The right panel shows the decoding accuracies for each of the 13 participants and the rightmost value indicates the average accuracy across the participants. Error bars indicate standard errors and a chance level is marked by the dashed line (20%). Note that the data from participants 6, 7, and 10 were excluded from the analysis.

A random-effects group analysis (N = 13) revealed that four neural clusters exhibited above-chance decoding accuracy results of discriminating five roughness levels (p < 0.001 uncorrected, cluster size > 30) (Fig 2 and Table 2). These four clusters were located in the supplementary motor area (SMA), the contralateral postcentral gyrus (S1), and the superior portions of the bilateral temporal pole (STP), respectively. The clusters that we found were unlikely to have occurred by chance: a bootstrap procedure [25] revealed that the probabilities of obtaining a cluster as large as ours were less than 5%. Therefore, our clusters remained significant after the correction for multiple comparisons [25, 26]. Decoding accuracies from each significant cluster were obtained as follows (presented as mean ± standard deviation, highest and lowest accuracy for each cluster): 40.1 ± 4.6%, 47%, and 32% for the SMA cluster; 37.4 ± 4.5%, 46%, and 27% for the contralateral S1 cluster; 34.6 ± 4.2%, 41%, and 26% for the contralateral STP cluster; and 33.6 ± 4.3%, 40%, and 28% for the ipsilateral STP. A one sample t-test verified that these group-wise decoding accuracy results significantly exceeded the chance level for every cluster (SMA: t_12_ = 14.91, p < 0.01; contralateral S1: t_12_ = 9.51, p < 0.01; contralateral STP: t_12_ = 11.66, p < 0.01; ipsilateral STP: t_12_ = 15.85, p < 0.01). Furthermore, we measured the decoding accuracies of excluded participants (i.e., participants 6, 7, and 10) in the identified brain regions. Although these participants could not discriminate the degrees of roughness in the behavioral experiments, the decoding performances were significantly higher than the chance level (20%) across the brain regions (p < 0.05). However, their decoding performances were largely lower compared to data from the 13 participants (S1 Table).

**Table 2:** Significant clusters for roughness decoding (p < 0.001 uncorrected, cluster size > 30).

Brain Regions	Side	MNI Coordinates			Cluster Size	T	Z
		x	y	z			
**Supplementary Motor Area**	**R**	**2**	**0**	**56**	**191**	**6.44**	**4.16**
-	L	-8	-6	52		6.07	4.03
-	R	4	-10	54		5.71	3.90
**Postcentral Gyrus**	**L**	**-44**	**-28**	**62**	**52**	**6.18**	**4.07**
-	L	-40	-34	66		5.36	3.75
**Superior Temporal Pole**	**R**	**58**	**4**	**-8**	**40**	**6.07**	**4.03**
**Superior Temporal Pole**	**L**	**-38**	**12**	**-24**	**41**	**5.88**	**3.96**

Side indicates hemisphere (R = right, L = left), cluster size indicates N voxels, T indicates peak t-values, Z indicates peak z-values. Entries without the brain region name-labels indicate sub-peaks within the cluster named above them.

**Fig 3:**
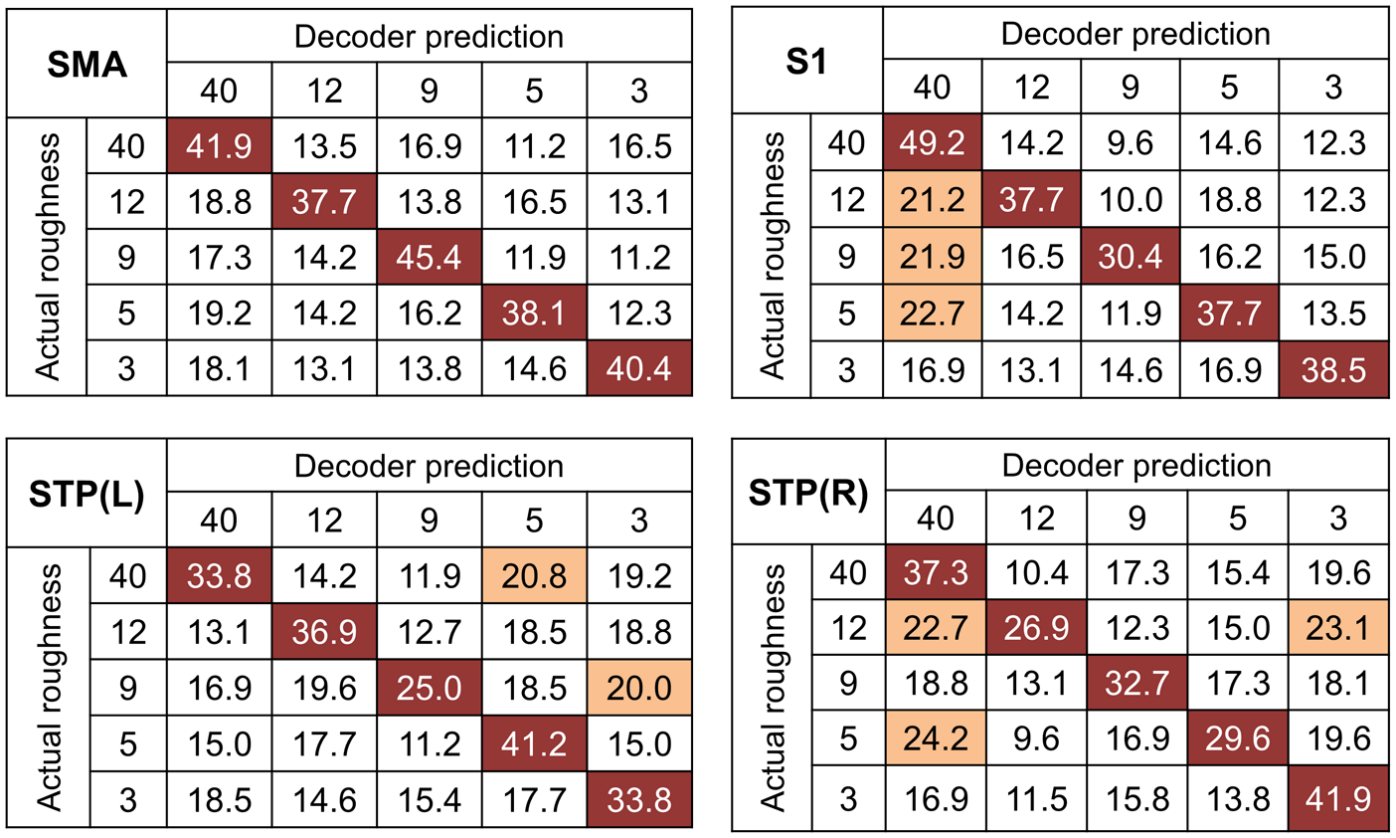
Confusion matrices for decoder predictions of fMRI activity in the significant clusters. The rows of the matrix indicate the actual roughness provided to the participants and the columns indicate the predictions by a neural decoder. The cells of highest accuracy in each row are highlighted in red and the frequent confusions, of which the misclassification rates exceeded the chance level (20%), are highlighted in light red.

Fig 3 shows the confusion matrices for each cluster. Note that the value on a row *i* and column *j* in each matrix represents the probability that a presentation of roughness *i* was predicted as roughness *j* (an ideal confusion matrix would have a 100% probability everywhere on the diagonal and 0% in the off-diagonal entries). In the confusion matrices, the highest accuracy and the frequent confusions (when the misclassification rate surpasses the chance level of 20%) are highlighted for each row (for each roughness level). In all four confusion matrices, the highest classification accuracy was always found on the diagonal entries. The highest performance of the GNB classifier was found with 49.2% accuracy using the values of S1, when a stimulus of particle size ‘40’ was provided. The lowest classification performance with 25.0% was found in the contralateral STP, when a stimulus of particle size ‘9’ was provided. Several specific patterns in the confusion matrices are also notable. First, the SMA was the only region that did not exhibit frequent confusion. It also showed the least variance of correct classification rates along the diagonal entries among the four regions: variance across the diagonal entries was 9.85%, 45.29%, 35.31%, and 36.06% for the SMA, S1, contralateral STP, and ipsilateral STP, respectively. Second, the misclassification rates were evenly distributed over the off-diagonal entries. We expected that the misclassification would tend towards a similar roughness level, but such a tendency was not observed.

The whole brain univariate GLM group analysis contrasting tactile exploration against resting periods identified activation clusters consistently in the primary visual cortex (V1), contralateral primary motor cortex (M1), and contralateral S1 (p < 0.001 uncorrected, cluster size > 100) across the five different roughness levels (see S2–S6 Tables). Taken together, the univariate analysis revealed distinct clusters mainly in the contralateral S1, contralateral M1, and V1, whereas the MVPA for stimulus roughness information identified significant clusters in the SMA, contralateral S1, and bilateral STP.

### Correlation Analysis

**Fig 4:**
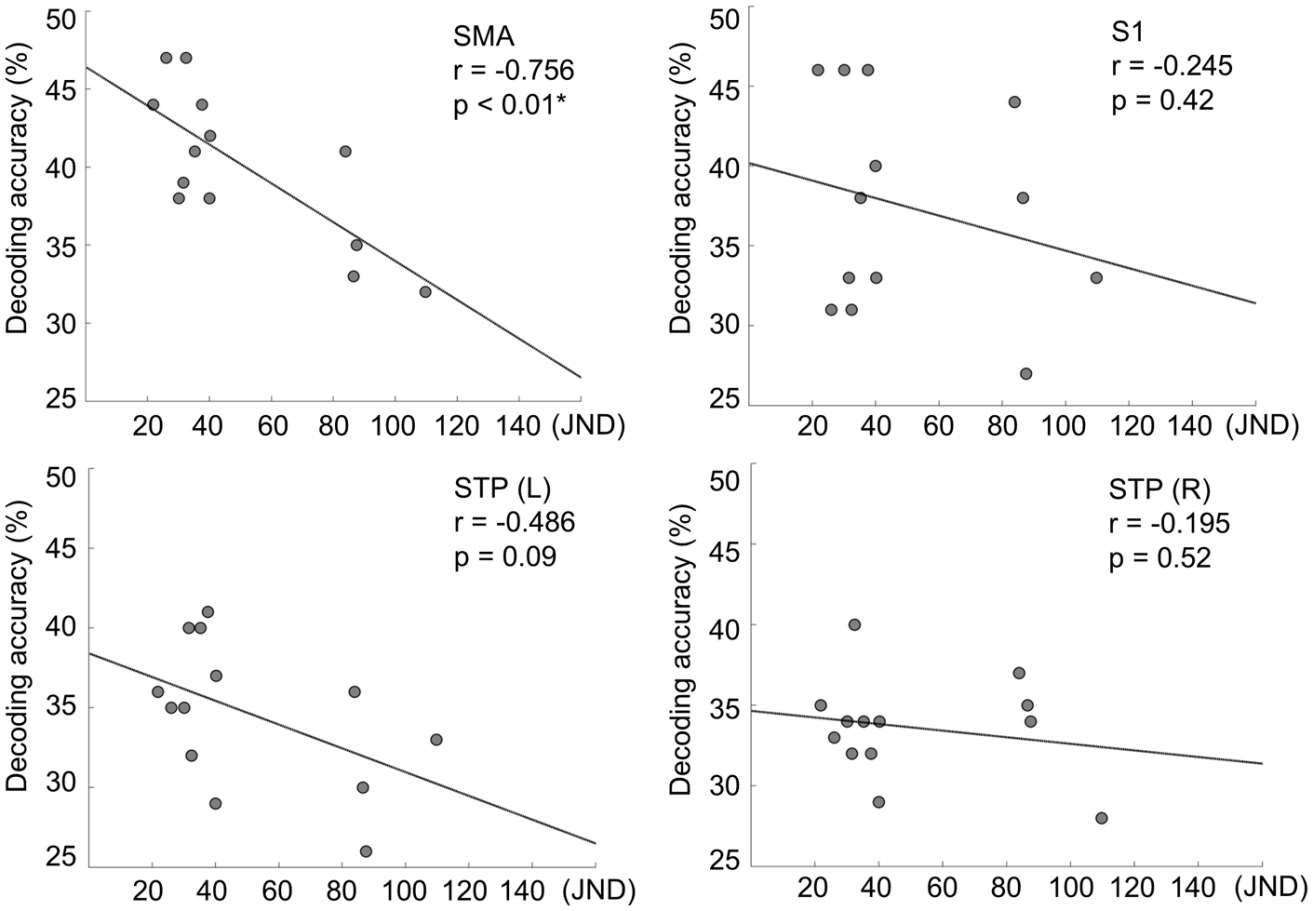
Correlations between behavioral and neural decoding accuracies for roughness discrimination. The 13 participants’ neural decoding accuracies are plotted over the JND values in the behavioral experiments. The solid lines show robust fits of linear relationships.

Having found specific brain regions that provided useful information for roughness classification as presented above, we investigated how regional classification accuracy of individual participants varied with their perceptual roughness discriminative sensitivity (JND). Data from 13 participants were used in this analysis; data from three were excluded (participants 6, 7, and 10). The pairwise correlation analysis revealed a significant correlation between JND and decoding accuracy in the SMA (r = -0.756, p < 0.01), but not in other regions: contralateral S1 (r = -0.245, p = 0.42), contralateral STP (r = -0.486, p = 0.09), and ipsilateral STP (r = -0.195, p = 0.52) (Fig 4). The negative correlation in the SMA indicated that a higher decoding accuracy from the SMA was obtained in those participants who showed a smaller JND value (i.e., better roughness discrimination). Another correlation analysis of decoding accuracy with percentage of correct answers did not show any significant correlation for all the searchlight clusters (all r < 0.3, p > 0.32).

## Discussion

In the present study, we demonstrated the feasibility of decoding the tactile information about degrees of roughness from brain activity using a searchlight MVPA. In particular, the SMA, the contralateral S1, and the bilateral STP appeared to carry useful information for roughness discrimination. In addition, we derived JNDs from the psychometric function for evaluating each participant’s perceptual sensitivity to tactile roughness and correlated them with the neural decoding accuracies resulting from the brain regions explored above. A significant correlation was found only in the SMA. It is noteworthy that SMA exhibited not only a correlation between the JNDs and decoding accuracies but also provided the most accurate and reliable decoding results. To our knowledge, this is the first attempt to show that neural decoding performance can predict individual perceptual sensitivity to tactile roughness.

It is well-established that the SMA is implicated in the preparation and execution of voluntary movements. Particularly, several studies have suggested that differences in movement intensity and speed during the active tactile exploration task could evoke distinct patterns of SMA activity [27–29]. For instance, Simoes-Franklin and colleagues considered significant SMA activities to be the reflection of the motor components of the active task, rather than tactile roughness categorization *per se* [29]. According to their consideration, in our fMRI data analysis, the fact that participants actively explored the tactile surfaces with their finger during the brain signal acquisition could potentially affect the multi-voxel response patterns in SMA. However, the significant SMA activation was observed during passive tactile discrimination tasks as well [30], indicating its potential role in tactile discrimination. Therefore, our observation lends support to the conjecture that the SMA may carry essential information for the differentiation of tactile roughness.

One of the key findings in the present study is the significant correlation between behavioral performance of tactile discriminability and neural decoding accuracy in the SMA. Previous studies in primates reported that neuronal activity in the SMA are associated with behavioral performance [31–33]. For example, Romo and Salinas reported that the neural activation patterns in the S1 reflect the physical properties of the tactile stimulation, regardless of how the monkeys perceived the stimulus, whereas neural activation patterns in the SMA were more closely correlated with their behavioral responses [33]. What then, could be a possible explanation for this correlation between SMA activity and behavioral performance (i.e., perceptual discriminability in our case)? An explanation may be provided in terms of how the somatosensory system achieves a neural representation of roughness. Tactile sensation is related to the variation in sensory afferent firing rates, with each afferent delivering information about a different texture or tactile property [34, 35]. In particular, the neural activity of roughness perception are modulated by the physical properties of the touched surface (e.g., the spatial distribution, height, and diameter of the grits), and it has been suggested that temporal characteristics of stimulation are important in the perception of tactile roughness [36–38]. Our results suggest that such differentiable neural representations of temporal characteristics may be exhibited most saliently in the SMA. Several primate studies support such a role of the SMA in representing temporal aspects of stimuli. For instance, using electrophysiological recordings in monkeys, Mita and colleagues found that neurons in the SMA and pre-SMA encode the information of time intervals between finger movements [39]. It was also shown that neurons in the SMA encode the memorized stimulus frequency and generate a neural signal correlating with the output of the animal’s decision [40]. Similarly, lesions and neuroimaging studies in humans demonstrated that SMA is engaged in both perceptual and motor timing processing [41–43].

Our results showed that the STP is also involved in the discrimination of tactile roughness levels. While the STP is widely known for auditory and language processing, several studies have suggested that the temporal lobe may also be involved in somatosensory functions, based on the fact that both auditory and tactile sensations rely on the transduction of physical events into neural coding of frequency [44]. Vibrotactile stimuli consistently activated the superior temporal gyrus together with the secondary somatosensory cortex [45] and temporal lobe epilepsy patients were severely impaired in a tactile grating orientation discrimination task [46]. The left superior temporal gyrus was activated by active and passive dynamic touches during a roughness categorization task [29].

A large number of studies have shown the involvement of S1 in tactile discrimination. Animal studies using single- and multi-unit recordings have shown that S1 neurons were able to reliably encode distinct frequency and texture stimuli [47, 48]. Human studies reported activation in the S1 during a roughness estimation task in a PET study [49, 50] and an fMRI study [51]. Our present results are consistent with these findings on tactile roughness information processing in the S1. However, it is rather unexpected that S1 activity did not correlate with individual perceptual sensitivity to roughness across participants despite its involvement in a variety of tactile roughness tasks.

It is worth noting that brain regions activated by the searchlight MVPA and the GLM analyses did not coincide; the S1 area was found to be significant in the searchlight MVPA and the GLM analyses, the SMA and STP areas were identified only in the searchlight MVPA, and the M1 area was identified only in the GLM analysis. This discrepancy could be explained by the motor involvement. On the one hand, the GLM analysis contrasted roughness exploration against resting periods. Since there were additional finger movements for stimulus exploration during the exploration period, GLM results can be influenced by motor information as well as tactile information. On the other hand, the searchlight analysis decoded five level of roughness information using fMRI data elicited solely during the roughness exploration periods, not the resting periods. Moreover, our fMRI experiments were designed to minimize the effects of individual differences of finger movements. Participants were instructed to explore the tactile surface using horizontal movements; the participants moved their right index fingertip from side to side. Since the size of the abrasive paper was relatively small (3 × 3 cm^2^), we assumed that all participants explored the provided stimulus in a similar manner throughout the experiment. Therefore, all input data to searchlight MVPA involved similar movements and we supposed that the classifier was unlikely to be influenced by the differences in finger movements. Taken together, the searchlight MVPA identified brain areas (e.g., the SMA, S1, and STP) exhibiting distinct roughness information and the GLM analysis identified brain areas (e.g., S1 and M1) exhibiting both motor and tactile roughness information. Our results thus support the following hypotheses: The SMA and STP encode different levels of roughness information independent of motor information, S1 plays a role in both motor and roughness information processing, and M1 activation reflects only the motor information.

We investigated cortical activation patterns with respect to the contrast of tactile exploration against resting periods. The univariate GLM group analysis identified activated clusters in V1, contralateral M1, and contralateral S1. It is not surprising that S1 and M1, the primary areas for sensorimotor processing, were activated by tactile exploration. The presence of S1 and M1 activities is in line with previous findings showing that these regions are engaged in tactile exploration [52]. A rather unexpected observation of activated clusters in V1 might be partly attributed to differences in visual instructions. During image acquisition, a fixation cross, ‘+’, was presented for the resting period and a Korean word, ‘자극’ (meaning ‘Stimulation’ in English), was shown for the exploration period on the screen. A previous fMRI study demonstrated that even subtle changes in visual stimuli could be identified from the BOLD signals in V1 [53]. Similarly, contrasting V1 activity between exploration and resting might capture distinct BOLD signals generated by different visual stimuli. However, further investigation is needed to verify this V1 activity differentiation with a fixation cross and a visual stimulus cue.

Since there is a clear relationship between age and tactile perceptual sensitivity [2, 54], we examined whether there were any effects on perceptual tactile sensitivity of gender or age. We found no significant influence on tactile sensitivity of either factor: gender (r = -0.099, p = 0.72) and age (r = -0.038, p = 0.89). Hence, our results suggest that the individual perceptual sensitivity to roughness observed in our study is related to brain properties independent of gender and age.

Our findings may be limited by the fact that participants did not perform any discrimination tasks during the fMRI experiments, in contrast to the behavioral experiments. In this respect, it is unclear if our correlation analysis between perceptual and neural discriminative performance was the most appropriate method. Another concern is an unusual method used in psychophysical experiment: We did not use a standard stimulus surface as reference. To partially solve this issue, we had performed the additional correlation analysis with percentage of correct answers, but no significant correlation was found. Thus, we cannot rule out the possibility of unexpected bias to the reported data due to non-linear roughness perception. Lastly, it should be stressed that we cannot assume the human brain to use the same tactile information as the neural decoder built in our study in order to discriminate roughness. Since this study mainly focuses on the feasibility of decoding tactile roughness information and correlation between neural decoding results and behavioral performance, we have not yet explored optimizing decoder parameters. As such, the SMA may not be the only brain region underlying individual variations of perceptual sensitivity to tactile roughness. Hence, it will be worth investigating these aspects in further studies.

## Conclusions

In this study, we statistically assessed each set of voxel response patterns across the whole brain and revealed that the SMA, the contralateral S1, and the bilateral STP exhibit neural activity patterns specific to roughness discrimination. We observed that the SMA showed a significant correlation of the behavioral and neural decoding performances. In addition, the SMA contained the most accurate and reliable multi-voxel sets for tactile roughness decoding. Our findings suggest that the multi-voxel pattern of activity in the SMA is more closely related to the human behavior in a roughness discrimination task. More work will be needed to verify the role of these areas; however, we have provided fundamental evidence on brain regions that contribute to the successful decoding of tactile stimuli. In addition, these results could motivate subsequent studies to examine the role of individual brain regions in tactile processing from the perspective of human behavior.

## Supporting Information

S1 Dataset Searchlight MVPA results for each of the 16 participants. Each matrix shows classification accuracies across the whole brain voxels.
(ZIP)

S1 Fig Examples of the psychometric function of the participant having (A) the best JND (participant 4) and (B) the worst JND (participant 6). Steeper slopes indicate that the participant was better able to discriminate between smaller differences of roughness intensity.
(TIF)

S1 Table Decoding accuracies for each of the excluded participants in the searchlight brain regions. (DOCX)

S2 Table Activated brain regions contrasting tactile exploration (particle size, 3 μm) against resting periods using a univariate GLM (p < 0.001 uncorrected, cluster size > 100). Side indicates hemisphere (R = right, L = left), cluster size indicates N voxels, T indicates peak t-values, Z indicates peak z-values.
(DOCX)

S3 Table Activated brain regions contrasting tactile exploration (particle size, 5 μm) against resting periods using a univariate GLM (p < 0.001 uncorrected, cluster size > 100). Side indicates hemisphere (R = right, L = left), cluster size indicates N voxels, T indicates peak t-values, Z indicates peak z-values.
(DOCX)

S4 Table Activated brain regions contrasting tactile exploration (particle size, 9 μm) against resting periods using a univariate GLM (p < 0.001 uncorrected, cluster size > 100). Side indicates hemisphere (R = right, L = left), cluster size indicates N voxels, T indicates peak t-values, Z indicates peak z-values.
(DOCX)

S5 Table Activated brain regions contrasting tactile exploration (particle size, 12 μm) against resting periods using a univariate GLM (p < 0.001 uncorrected, cluster size > 100). Side indicates hemisphere (R = right, L = left), cluster size indicates N voxels, T indicates peak t-values, Z indicates peak z-values.
(DOCX)

S6 Table Activated brain regions contrasting tactile exploration (particle size, 40 μm) against resting periods using a univariate GLM (p < 0.001 uncorrected, cluster size > 100). Side indicates hemisphere (R = right, L = left), cluster size indicates N voxels, T indicates peak t-values, Z indicates peak z-values.
(DOCX)
