# Supplementary figures and images for: Decoding Accuracy in Supplementary Motor Cortex Correlates with Perceptual Sensitivity to Tactile Roughness
Source: PLoS One. 2015 Jun 11;10(6):e0129777. doi: 10.1371/journal.pone.0129777 (PMC4465937; doi:10.1371/journal.pone.0129777)

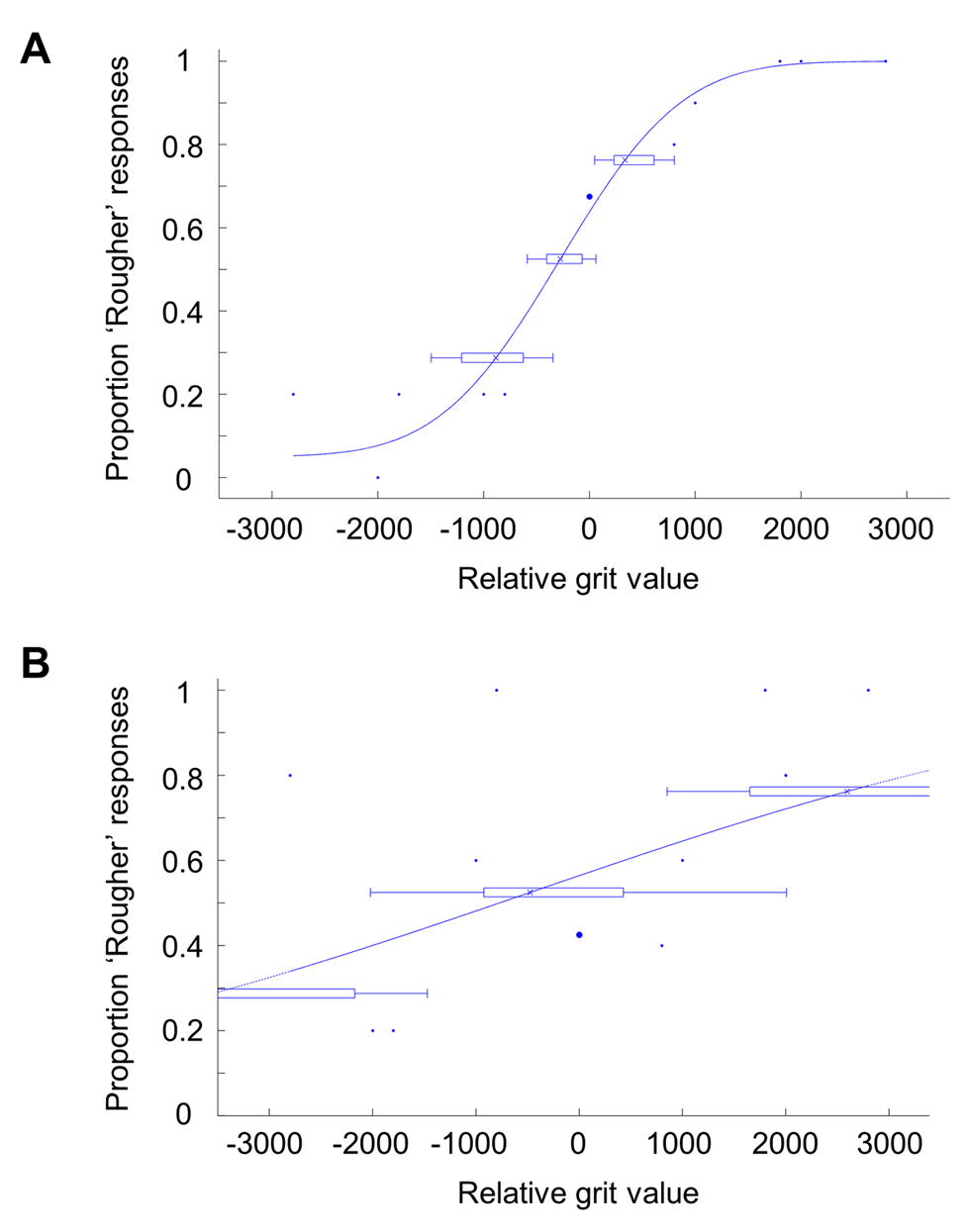

Supplement: S1 Fig — Steeper slopes indicate that the participant was better able to discriminate between smaller differences of roughness intensity. (TIF) [file pone.0129777.s002.tif]
