# Supplementary material for: Decoding Accuracy in Supplementary Motor Cortex Correlates with Perceptual Sensitivity to Tactile Roughness
Source: PLoS One. 2015 Jun 11;10(6):e0129777. doi: 10.1371/journal.pone.0129777 (PMC4465937; doi:10.1371/journal.pone.0129777)
Supplement: S1 Table — (DOCX) [file pone.0129777.s003.docx]

**S1 Table.**

|  | Participant 6 | Participant 7 | Participant 10 |
| --- | --- | --- | --- |
| SMA | 29.8 ± 2.3 % | 25.5 ± 3.1 % | 30.2 ± 2.3 % |
| S1 | 29.1 ± 3.6 % | 30.0 ± 2.2 % | 31.6 ± 2.9 % |
| STP (L) | 24.3 ± 2.6 % | 30.6 ± 2.2 % | 26.2 ± 3.2 % |
| STP (R) | 25.6 ± 2.2 % | 29.3 ± 2.8 % | 24.9 ± 3.2 % |
