# Supplementary material for: Decoding Accuracy in Supplementary Motor Cortex Correlates with Perceptual Sensitivity to Tactile Roughness
Source: PLoS One. 2015 Jun 11;10(6):e0129777. doi: 10.1371/journal.pone.0129777 (PMC4465937; doi:10.1371/journal.pone.0129777)
Supplement: S2 Table — Side indicates hemisphere (R = right, L = left), cluster size indicates N voxels, T indicates peak t-values, Z indicates peak z-values. (DOCX) [file pone.0129777.s004.docx]

**S2 Table.**

| Brain Regions | Side | MNI coordinates | | | Voxels | T | Z |
| --- | --- | --- | --- | --- | --- | --- | --- |
|  |  | x | y | z |  |  |  |
|  |  |  |  |  |  |  |  |
| **Lingual gyrus** | **R** | **15** | **-85** | **-11** | **2118** | **11.73** | **5.82** |
| Fusiform gyrus | R | 24 | -79 | -11 |  | 11.37 | 5.75 |
| Middle occipital gyrus | L | -15 | -103 | 1 |  | 9.72 | 5.38 |
|  |  |  |  |  |  |  |  |
| **Precentral gyrus** | **L** | **-36** | **-19** | **52** | **316** | **6.25** | **4.32** |
| Postcentral gyrus | L | -54 | -16 | 52 |  | 5.75 | 4.12 |
| Precentral gyrus | L | -42 | -7 | 61 |  | 5.57 | 4.04 |
|  |  |  |  |  |  |  |  |
