# Supplementary material for: Decoding Accuracy in Supplementary Motor Cortex Correlates with Perceptual Sensitivity to Tactile Roughness
Source: PLoS One. 2015 Jun 11;10(6):e0129777. doi: 10.1371/journal.pone.0129777 (PMC4465937; doi:10.1371/journal.pone.0129777)
Supplement: S3 Table — Side indicates hemisphere (R = right, L = left), cluster size indicates N voxels, T indicates peak t-values, Z indicates peak z-values. (DOCX) [file pone.0129777.s005.docx]

**S3 Table.**

| Brain Regions | Side | MNI coordinates | | | Voxels | T | Z |
| --- | --- | --- | --- | --- | --- | --- | --- |
|  |  | x | y | z |  |  |  |
|  |  |  |  |  |  |  |  |
| **Middle occipital gyrus** | **L** | **-18** | **-100** | **4** | **1594** | **8.97** | **5.20** |
| Cuneus | R | 12 | -100 | 7 |  | 8.59 | 5.09 |
| Calcarine | L | -15 | -97 | -5 |  | 8.46 | 5.06 |
|  |  |  |  |  |  |  |  |
| **Precentral gyrus** | **L** | **-39** | **-10** | **64** | **350** | **7.93** | **4.90** |
| Postcentral gyrus | L | -54 | -16 | 52 |  | 7.82 | 4.87 |
| Precentral gyrus | L | -36 | -19 | 61 |  | 6.70 | 4.49 |
|  |  |  |  |  |  |  |  |
