# Supplementary material for: Decoding Accuracy in Supplementary Motor Cortex Correlates with Perceptual Sensitivity to Tactile Roughness
Source: PLoS One. 2015 Jun 11;10(6):e0129777. doi: 10.1371/journal.pone.0129777 (PMC4465937; doi:10.1371/journal.pone.0129777)
Supplement: S4 Table — Side indicates hemisphere (R = right, L = left), cluster size indicates N voxels, T indicates peak t-values, Z indicates peak z-values. (DOCX) [file pone.0129777.s006.docx]

**S4 Table.**

| Brain Regions | Side | MNI coordinates | | | Voxels | T | Z |
| --- | --- | --- | --- | --- | --- | --- | --- |
|  |  | x | y | z |  |  |  |
|  |  |  |  |  |  |  |  |
| **Middle occipital gyrus** | **L** | **-21** | **-100** | **10** | **2082** | **11.68** | **5.81** |
| Middle occipital gyrus | L | -39 | -79 | -23 |  | 9.85 | 5.42 |
| Middle occipital gyrus | L | -30 | -88 | -23 |  | 9.83 | 5.41 |
|  |  |  |  |  |  |  |  |
| **Precentral** **gyrus** | **L** | **-42** | **-7** | **61** | **253** | **6.40** | **4.38** |
| Precentral gyrus | L | -36 | -19 | 55 |  | 6.38 | 4.37 |
| Precentral gyrus | L | -36 | -16 | 54 |  | 6.20 | 4.30 |
|  |  |  |  |  |  |  |  |
