# Supplementary material for: Decoding Accuracy in Supplementary Motor Cortex Correlates with Perceptual Sensitivity to Tactile Roughness
Source: PLoS One. 2015 Jun 11;10(6):e0129777. doi: 10.1371/journal.pone.0129777 (PMC4465937; doi:10.1371/journal.pone.0129777)
Supplement: S5 Table — Side indicates hemisphere (R = right, L = left), cluster size indicates N voxels, T indicates peak t-values, Z indicates peak z-values. (DOCX) [file pone.0129777.s007.docx]

**S5 Table.**

| Brain Regions | Side | MNI coordinates | | | Voxels | T | Z |
| --- | --- | --- | --- | --- | --- | --- | --- |
|  |  | x | y | z |  |  |  |
|  |  |  |  |  |  |  |  |
| **Lingual gyrus** | **R** | **15** | **-88** | **-11** | **1902** | **10.54** | **5.57** |
| Lingual gyrus | R | 21 | -79 | -11 |  | 8.92 | 5.18 |
| Superior occipital gyrus | R | 18 | -106 | 7 |  | 8.83 | 5.16 |
|  |  |  |  |  |  |  |  |
| **Precentral gyrus** | **L** | **-36** | **-13** | **64** | **223** | **7.04** | **4.61** |
| Precentral gyrus | L | -39 | -13 | 64 |  | 6.08 | 4.25 |
| Precentral gyrus | L | -30 | -22 | 64 |  | 5.45 | 3.99 |
|  |  |  |  |  |  |  |  |
| **Supplementary motor area** | **L** | **0** | **5** | **49** | **129** | **6.51** | **4.42** |
| Supplementary motor area | R | 3 | 2 | 61 |  | 6.32 | 4.35 |
| Supplementary motor area | L | -3 | 2 | 73 |  | 5.05 | 3.80 |
|  |  |  |  |  |  |  |  |
