# Supplementary material for: Decoding Accuracy in Supplementary Motor Cortex Correlates with Perceptual Sensitivity to Tactile Roughness
Source: PLoS One. 2015 Jun 11;10(6):e0129777. doi: 10.1371/journal.pone.0129777 (PMC4465937; doi:10.1371/journal.pone.0129777)
Supplement: S6 Table — Side indicates hemisphere (R = right, L = left), cluster size indicates N voxels, T indicates peak t-values, Z indicates peak z-values. (DOCX) [file pone.0129777.s008.docx]

**S6 Table.**

| Brain Regions | Side | MNI coordinates | | | Voxels | T | Z |
| --- | --- | --- | --- | --- | --- | --- | --- |
|  |  | x | y | z |  |  |  |
|  |  |  |  |  |  |  |  |
| **Middle occipital gyrus** | **L** | **-18** | **-97** | **-2** | **2018** | **9.96** | **5.44** |
| Lingual gyrus | R | 15 | -85 | -5 |  | 8.63 | 5.10 |
| Middle occipital gyrus | L | -21 | -103 | 4 |  | 8.49 | 5.06 |
|  |  |  |  |  |  |  |  |
| **Postcentral gyrus** | **L** | **-54** | **-16** | **52** | **294** | **7.51** | **4.77** |
| Precentral gyrus | L | -39 | -10 | 58 |  | 7.44 | 4.74 |
| Precentral gyrus | L | -30 | -13 | 70 |  | 6.04 | 4.24 |
|  |  |  |  |  |  |  |  |
| **Superior temporal gyrus** | **L** | **-51** | **11** | **-11** | **155** | **7.13** | **4.64** |
| Rolandic operculum | L | -48 | -4 | 4 |  | 6.44 | 4.40 |
| Superior temporal gyrus | L | -57 | 2 | -2 |  | 5.81 | 4.14 |
|  |  |  |  |  |  |  |  |
